# Correlation between abnormal energy metabolism of ovarian granulosa cells and in vitro fertilization–embryo transfer outcomes in patients with polycystic ovary syndrome and obesity

**DOI:** 10.1186/s13048-023-01204-3

**Published:** 2023-07-21

**Authors:** Ya-Kun Zhao, Ya-Na Gao, Ling-Chao Wang, Jing Wang, Gai-Jing Wang, Hong-Li Wu

**Affiliations:** 1 Department of Obstetrics and Gynecology, Affiliated Hospital of Hebei University, NO. 212 Yuhua East Road, Lianchi District, Baoding, 071000 Hebei China; 2 66350 Medical Company of PLA, Baoding, 071000 Hebei China

**Keywords:** Glycolysis, Granulosa cells, Mitochondrial functions, Polycystic ovary syndrome

## Abstract

**Context:**

Granulosa cells (GCs) that surround oocytes in mammalian reproduction play an active role in oocyte differentiation through proliferation and energy production.

**Aims:**

This study aimed to investigate the characteristics of the energy metabolism of ovarian GCs and the influence of GCs on the early embryonic development in polycystic ovary syndrome (PCOS).

**Methods:**

The clinical characteristics and in vitro fertilization-embryo transfer treatment outcomes of 39 patients with PCOS and 68 patients with simple tubal factor infertility who underwent controlled ovarian hyperstimulation were analyzed and summarized. The mitochondrial function and glucose metabolism level of the GCs were determined, as well as the content of oxidative stress markers in the follicular fluid (FF) of patients with and without PCOS.

**Key results:**

When compared to the non-PCOS group, patients with PCOS had a significantly increased number of retrieved oocytes but a significantly decreased number of high-quality embryos, available embryos, and high-quality blastocyst formation (*P* < 0.05). Furthermore, the mitochondrial membrane potential, adenosine triphosphate level, and mitochondrial DNA (mtDNA) copy number decreased in the GCs, whereas the levels of reactive oxygen species increased (*P* < *0.01*). The levels of malondialdehyde and 8-oxo-deoxyguanosine (8-OHdG) in the follicular fluid (FF) of the patients with PCOS were higher than those of the control group (*P* < *0.05)*, and superoxide dismutase was increased by compensation *(P* < *0.05).* In the PCOS group, the expressions of *GLUT1*, *LDHA*, and *PFKP* were lower than those in the non-PCOS group, and glucose levels were higher.

**Conclusions:**

The low oocyte competence of PCOS may be associated with mitochondrial dysfunction and abnormal glycolysis.

**Implications:**

This research offers explanations for the possible connections influencing human ovarian folliculogenesis.

## Introduction

The primordial follicle is the basic female reproduction organ and the only source of oocyte reserve. The development of follicles starts at the primordial stage and progresses through the preantral, antral, and preovulation stages. This process is characterized by follicular volume expansion and the proliferation and functional differentiation of granulosa cells (GCs). The process of maturation is lengthy, dynamic, and continuous. During oocyte maturation, fertilization, and embryonic development, a substantial amount of adenosine triphosphate (ATP) is produced by the oxidative phosphorylation of oocyte mitochondria to provide energy.

However, studies have demonstrated that oocytes have low glycolytic activity and preferentially use glucose uptake to obtain energy substrates from GCs for energy homeostasis^.^ [1]. One such example is pyruvate, a glycolysis pathway product of GCs, which is transported to the mitochondria of oocytes as a substrate for ATP energy production by oocytes through the monocarboxylic acid transport system. Therefore, in mammalian reproduction, the GCs surrounding the oocyte play an active role in oocyte differentiation and regulation through their proliferation and energy production [2, 3]. These functions can be dysregulated to cause severe cellular damage, resulting in decreased oocyte nuclear maturation and fertilization rates [4–6].

Polycystic ovary syndrome (PCOS) is one of the most prevalent female endocrine and metabolic disorders, affecting approximately 5%–10% of women of reproductive age [7, 8]. Data from several studies indicate that oocytes collected from patients with PCOS undergoing in vitro fertilization (IVF) are frequently of poor quality [9], resulting in a high cancellation rate and a low fertilization rate [10, 11]. The pathophysiology of the poor oocyte quality of PCOS is incompletely understood.

## Materials and methods

This experimental study was approved by the ethics committee of The Affiliated Hospital of Hebei University. All patients provided written informed consent, and their confidentiality and anonymity were protected. This study was registered at HDFY-LL-2022–081 and the registration number is HDFY-LL-2022–081 and conformed the Enhancing the QUAlity and Transparency Of health Research (EQUATOR) network guidelines.

### Participants

Samples were collected from patients with PCOS (the PCOS group) and patients with tubal factor infertility (the non-PCOS group) during their cycle of IVF-embryo transfer (IVF-ET). In the pre-ovulatory phase, a short-acting gonadotropin-releasing hormone agonist long protocol was employed. The range of reproductive ages was between 21 and 33 years. A total of 68 patients with tubal factor infertility and 39 patients with PCOS were enrolled in the present study.

The inclusion criteria were as follows: (1) diagnosis of PCOS: rare ovulation or anovulation (cycles longer than 35 days or shorter than 26 days); high androgen clinical manifestations or hyperandrogenism; polycystic ovaries (12 or fewer [2–9 mm] follicles in each ovary identified by transvaginal ultrasonography); (2) compliance with at least two of the above, excluding other diseases causing related symptoms; and (3) all patients without PCOS had normal ovarian morphology and regular menstrual cycles with tubal factor infertility.

The exclusion criteria were as follows: (1) the presence of endocrine or metabolic diseases; (2) a history of ovarian surgery or the presence of only one ovary; (3) a history of taking hormonal medication within the past three months; and (4) uterine malformations.

### Clinical and in vitro fertilization cycle characteristics

Before and during their first cycle of IVF-ET, clinical data and endocrine characteristics were collected retrospectively from patients with PCOS and tubal factor infertility. The total number of retrieved oocytes, the number of MII oocytes, the number of 2PN embryos, the overall fertilization rate, the high-quality embryo rate, the available embryo rate, the high-quality blastocyst formation rate, and the clinical pregnancy rate were evaluated to determine the outcome of the IVF cycles.

### Primary granulosa cell culture

Patients who underwent IVF-ET by controlled ovarian hyperstimulation had their mural GCs and follicular fluid (FF) extracted, isolated, and pooled. After the cumulus-oocyte complexes were removed from the follicular contents, the FF was collected and centrifuged at 450 × g for 3 min. The clear supernatant was stored at − 80 °C to assess the concentrations of glucose, pyruvate, and oxygen species.

The GCs were divided into two parts: one part was stored at − 80 °C for the detection of gene expression related to cell energy metabolism, while the other part was resuspended in 3 mL of culture medium (DMEM/F12, 100 IU/mL of penicillin, 0.1 mg/mL of streptomycin, and 10% fetal calf serum; Thermo Fisher Scientific, Inc., Waltham, MA, USA) and transferred to a 6-well culture dish at approximately 2.5 × 10^5^ cells/well. After 24 h, the attached cells were counted and seeded at densities of 2 × 10^4^ cells/well in a 96-well culture dish for 24 h, and the mitochondrial function and glycolysis of the cells were analyzed. The oocytes were then graded and inseminated. Pronuclear scoring and embryo quality were evaluated 16–18 h after insemination.

### Mitochondrial membrane potential level

The mitochondrial status of the GCs was determined using the lipophilic cation, 5,5’,6,6’-tetrachloro-1,1’,3,3’-tetraethylbenzimidazol carbocyanine iodide. Briefly, after 24 h of culture, the GCs from all the patients were attached, and a mitochondrial membrane potential (MMP) detection kit (Solarbio, Beijing, China) was used to detect the MMP. The relative MMP was then calculated using a fluorescence spectrophotometer (Olympus, Tokyo, Japan).

### Adenosine triphosphate content

The ATP content of the cultured GCs from the two groups was measured using a Firefly luciferase-based ATP kit (Beyotime, Nanjing, China) according to the instructions provided by the manufacturer. Briefly, the GCs were cultured at 2 × 10^4^ cells/well in 96-well plates for 24 h, lysed in an ATP lysis buffer (from the kit), and centrifuged at 12,000 × g for 10 min. The supernatants were then combined with the testing buffer, and the luminometer was used to measure the ATP concentrations. The experiments were conducted in triplicate.

### Intracellular reactive oxygen species production

2,7-dichlorofluorescein diacetate (DCFH-DA) (Solarbio, Beijing, China) is a cell-permeable, peroxide-sensitive fluorescent probe that was used to detect intracellular free radicals. Once DCFH-DA entered the cells and hydrolyzed to DCFH in the presence of free radicals such as H_2_O_2_ and peroxides, DCFH was oxidized to the green, fluorescent product dichlorofluorescein (DCF) and trapped in the cell compartment, where it could be measured by a fluorescence spectrophotometer. The wavelength of the laser was 488–525 nm (excitation and emission, respectively).

In the present study, two GC groups were collected and incubated with DCFH-DA at 37 °C for 20 min. Then, fluorescent DCF was measured using a fluorescence spectrophotometer, and the relative absorbance values that characterize the reactive oxygen species (ROS) content were measured.

### Extraction of DNA and quantification of mitochondrial DNA

According to the recommendations of the manufacturer, the total DNA extraction from isolated GC fractions was performed using a tissue genome DNA extraction kit (Shanghai, China). The mean mitochondrial DNA (mtDNA) copy number of the GCs was determined by real-time quantitative polymerase chain reaction (qPCR) using the SYBR® Green DNA intercalator on the Chromo4™ System (Bio-Rad, Hercules, CA, USA) in a 20-µL reaction volume containing a final concentration of 0.4 µM of each gene-specific primer and 1 µL of template. The mitochondrial gene quantitative primers were ND1 forward *5′-cctagccgtttactcaatcct-3′* and reverse *5′-tgatggctagggtgacttcat-3′*. The nuclear gene primers were β-actin forward *5′-tggcacccagcacaatgaa-3′* and reverse *5′-ctaagtcatagtccgcctagaagca-3′* as an internal control to quantitate the nuclear DNA in the GCs. Each sample was run in triplicate. The mtDNA copy number was calculated using the delta Ct (∆Ct) of the average Ct of the mtDNA and nuclear DNA (∆Ct = Ct × mtDNA – Ct × *β-actin*). The relative level of the mtDNA copy number was calculated using the 2^−∆∆Ct^ method.

### Activity of oxidative stress markers in the follicular fluid

The GCs were isolated from the aspirated FF in all patients using gradient centrifugation. The levels of malondialdehyde (MDA) and superoxide dismutase (SOD) in the FF were measured using thiobarbituric acid and the chemiluminescence technique, respectively. In the FF, *8-OHdG* was quantitatively detected by enzyme-linked immunosorbent assay (Jiancheng, Nanjing, China). The evaluations were repeated three times.

### Glucose test assay

According to the manufacturer’s protocol, the glucose levels of the FF in all patients were measured using a glucose assay kit (Jiancheng, Nanjing, China). The samples were measured using a microplate reader at an absorbance of 505 nm. The evaluations were repeated three times.

### Pyruvate production assay

The pyruvate concentrations of the FF in all patients were measured at an absorbance of 450 nm, according to the manufacturer’s protocol (Jiancheng, Nanjing, China). The experiments were conducted in triplicate.

### Reverse transcription-polymerase chain reaction

Total RNA was extracted from the GCs in all patients using TRIzol™ (Solarbio, Beijing, China). The complementary DNA (cDNA) was then synthesized using a RevertAid First Strand cDNA Synthesis Kit (Jierui, Shanghai, China). The mRNA expression of *GLUT1*/*LDHA*/*PFKP* was measured by reverse transcription-polymerase chain reaction (RT-PCR) using the Power SYBR® Green PCR Master Mix (Jierui, Shanghai, China) and normalized by the expression of the *β-actin* gene.

The primers were selected according to previous reports as follows: *GLUT1* forward *5'-ccagctgccattgccgtt-3’* and reverse *5'-gacgtagggaccacacagttgc-3'*; *LDHA* forward *5’-tgcacccagatttagggactgat-3’* and reverse *5’-cccaggatgtgtagcctttgag-3’*; *PFKP* forward *5’-aggcgatggacgagaggagat-3’* and reverse *5’-tgatggcaagtcgcttgtag-3’*. The specificity of these primers was validated by a dissociation curve analysis. The relative mRNA levels were expressed as 2^−∆∆Ct^ values.

### Statistical analysis

The data are presented as mean ± standard deviation. Statistical differences were analyzed via t-tests for the two groups. Analyses were performed using GraphPad Prism software version 5 (GraphPad Software, San Diego, CA, USA). A value of *P* < 0.05 was considered statistically significant.

## Results

### Characterization of the clinical in vitro fertilization cycle

There were statistically significant differences (*P* < 0.05) in body weight, fasting blood glucose, follicle-stimulating hormone (FSH), luteinizing hormone (LH), and testosterone (T) between the two groups. In the PCOS group, these indicators were higher than in the non-PCOS group under the same gonadotropin dosage and usage time. The number of retrieved oocytes in the PCOS group was significantly higher than that in the non-PCOS group, whereas the high-quality embryo rate, the available embryo rate, and the high-quality blastocyst formation rate decreased in the PCOS group (*P* < 0.05). However, the clinical pregnancy rate (%) in the PCOS group was 38.46%, and 77.14% in the non-PCOS group. Tables 1 and 2 depicts the general information and specific data of the IVF–ET of the two groups.

**Table 1:** Comparison of patients’ characteristics in both groups (mean ± standard deviation)

Parameters	PCOS	Control	*P* value	95% Confidence Interval
Number of cases	39	68		
Age(years)	28.6 ± 0.49	29.5 ± 0.34	0.15	(-2.04, 0.30)
BMI (kg/m2)	26.1 ± 0.56	22.5 ± 0.43	< 0.01**	(2.23, 5.06)
Duration of infertility (years)	3.4 ± 1.0	3.5 ± 1.80	0.99	(0.02, 0.61)
Fasting blood glucose	5.5 ± 0.21	5.1 ± 0.06	0.03*	(0.04, 0.74)
base-FSH (mIU/ml)	5.54 ± 0.27	4.70 ± 0.17	0.05*	(-1.58, -0.09)
base-LH (mIU/ml)	3.80 ± 0.27	7.00 ± 0.49	0.01**	(2.17, 4.23)
base-T (nmol/l)	0.98 ± 0.04	1.41 ± 0.11	0.01**	(0.23, 0.61)

*p < 0.05

**p < 0.01

**Table 2:** IVF outcome parameters in both groups (mean ± standard deviation)

Parameters	PCOS	Control	*P* value	95% confidence interval
Retrieved oocytes	15.92 ± 0.88	13.74 ± 0.64	0.04*	(0.05, 4.32)
Number of MII	14.49 ± 0.83	12.59 ± 0.57	0.06	(-0.05, 3.84)
2PN of fertilization	11.51 ± 0.72	10.01 ± 0.47	0.07	(-0.13, 3.12)
Top quality embryos	5.15 ± 0.51	5.12 ± 0.33	0.95	(-1.11, 1.19)
Available embryos	7.03 ± 0.57	6.97 ± 0.39	0.93	(-1.27, 1.38)
Fertilization rate (%)	72.3	72.91	0.82	
High quality embryos rate (%)	45.07	51.4	0.04*	
Available embryos rate (%)	50.37	58.45	0.01**	
High quality blastocyst formation rate (%)	10.09	14.18	0.04*	
Clinical pregnancy rate (%)	38.46	77.14	0.02*	

*p < 0.05

**p < 0.01

### Mitochondrial membrane potential levels

**Fig. 1:**
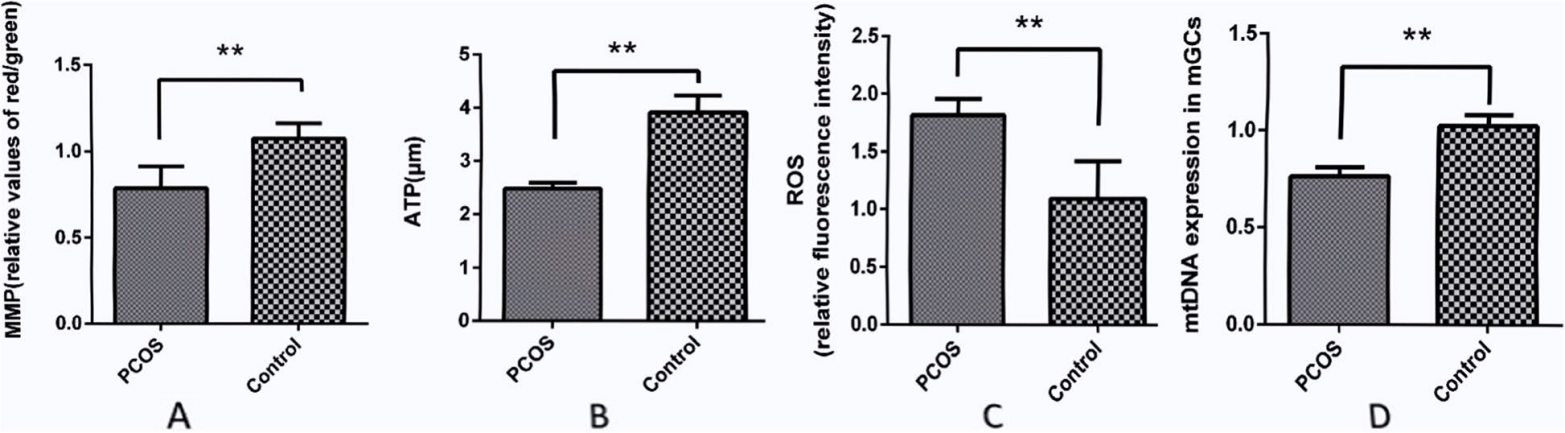
The measurements of mitochondrial membrane potential (MMP), adenosine triphosphate (ATP), reactive oxygen species (ROS), and mitochondrial DNA copy number. MMP in human granulosa cells (GCs) was compared between patients with and without polycystic ovary syndrome, measured by the ratio of red to green fluorescence intensity. The bars represent the mean ± standard deviation of MMP in 2 × 10^4^ GCs (**A**). ATP content in human granulosa cells (GCs) was compared and measured between patients with and without polycystic ovary syndrome. The bars represent the mean ± standard deviation of ATP content in 2 × 10^4^ GCs (**B**). The level of ROS in human granulosa cells (GCs) from patients with and without polycystic ovary syndrome (PCOS), as measured by fluorescence spectrophotometer. The average fluorescence intensity of non-PCOS GCs was defined as 1.0. The fluorescence intensity of ROS was normalized by non-PCOS in 2 × 10.^4^ GCs (**C**). The relative level of mitochondrial DNA copy number in human granulosa cells from patients with and without polycystic ovary syndrome was measured by RT-PCR. Data are presented as the mean ± standard deviation. A t-test was used for statistical analysis: **, *P* < 0.01. The assays were repeated three times (**D**)

In this study, the GCs were collected from the mature follicles of non-PCOS and PCOS ovaries undergoing ovarian stimulation for IVF, and the MMP was compared between these two groups. The MMPs of the GCs were analyzed using image-based cytometry, as shown in Fig. 1A. The MMP of the GCs was significantly lower in the PCOS group in comparison with the non-PCOS group (0.785 ± 0.04 vs. 1.073 ± 0.09, respectively).

### Adenosine triphosphate content

In this study, the GCs were collected from the mature follicles of non-PCOS and PCOS ovaries undergoing ovarian stimulation for IVF, and the ATP was compared between these two groups. Figure 1B shows the ATP content of the GCs was significantly lower in the PCOS group than in the non-PCOS group (2.484 ± 0.03 µmol vs. 3.916 ± 0.09 µmol, respectively; *P* < 0.01).

### Reactive oxygen species levels

In this study, the GCs were collected from matured follicles of non-PCOS and PCOS ovaries undergoing ovarian stimulation for IVF, and the ROS levels were compared between these two groups. The ROS levels of the GCs obtained from the PCOS group significantly increased by 1.9 times (*P* < 0.01) when compared with the non-PCOS group, as shown in Fig. 1C.

### The mitochondrial DNA copy number in granulosa cells

In this study, the GCs were collected from the mature follicles of non-PCOS and PCOS ovaries undergoing ovarian stimulation for IVF, and the mtDNA levels were compared between these two groups. Figure 1D shows the relative mtDNA levels quantified by RT-PCR were significantly decreased in the GCs obtained from patients with PCOS when compared with those in the non-PCOS group (0.761 ± 0.05 vs. 1.021 ± 0.06, respectively; *P* = 0.01).

### Oxidative stress markers in follicular fluid

**Fig. 2:**
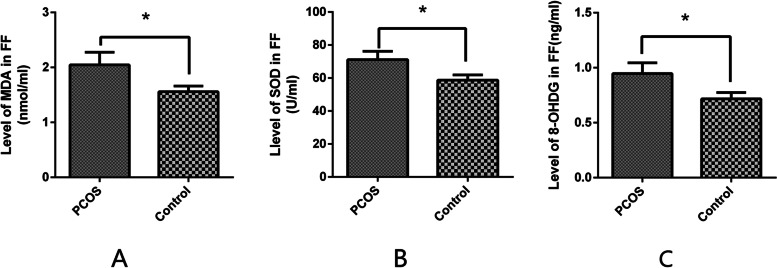
The status of peroxidation and antioxidants in follicular fluid from patients with and without polycystic ovary syndrome was evaluated using malondialdehyde (**A**), superoxide dismutase (**B**), and *8-OHdG* (**C**), which were detected using appropriate experimental kits. Data are presented as the mean ± standard deviation. A t-test was used for statistical analysis: *, *P* < 0.05. The assay was repeated three times

The levels of MDA, SOD, and *8-OHdG* in the FF detected via the relevant experimental kits significantly increased when compared with the non-PCOS group and were accompanied by a decrease in mtDNA levels in patients with PCOS (MDA: 1.558 ± 0.10 vs. 2.047 ± 0.23 nmol/mL; SOD: 58.71 ± 3.13 vs. 71.17 ± 5.03 U/mL; *8-OHdG*: 0.7163 ± 0.06 vs. 0.9469 ± 0.10 ng/mL) (Figs. 2A–C).

### Expression of the glycolysis-associated gene in granulosa cells

**Fig. 3:**
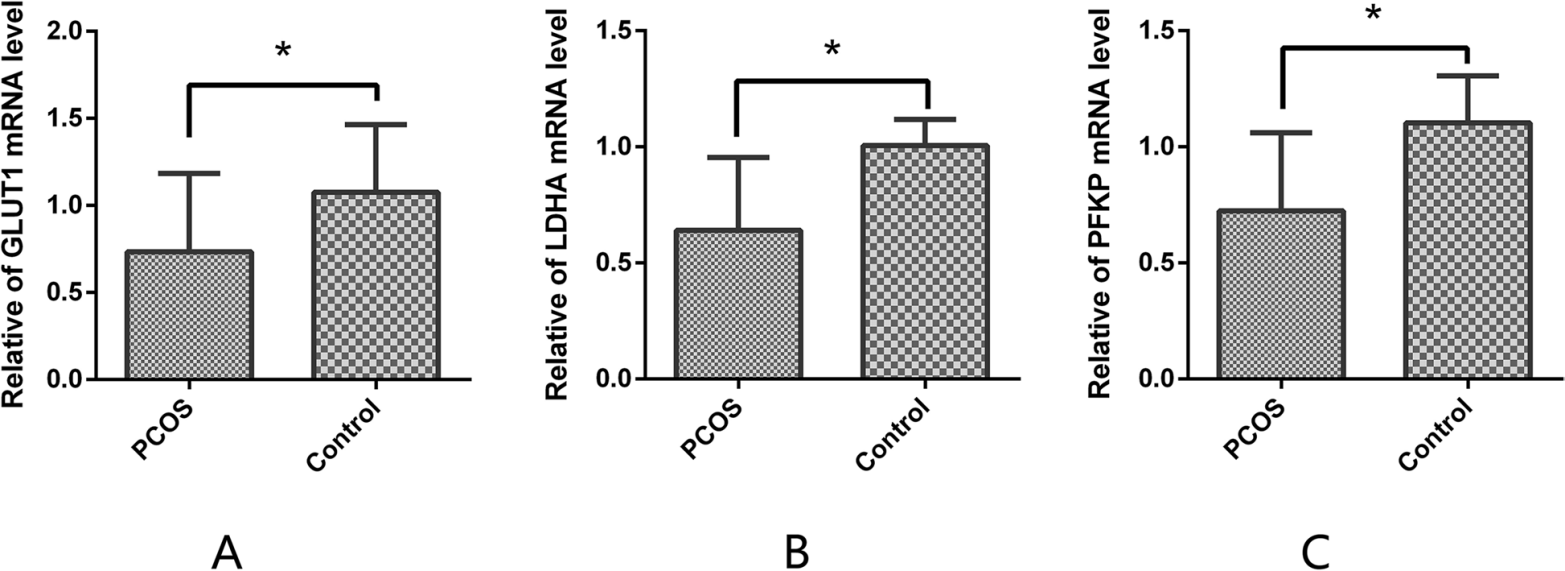
The expressions of glycolysis-associated genes *GLUT1* (**A**), *LDHA* (**B**), and *PFKP* (**C**) in the granulosa cells of patients with and without polycystic ovary syndrome were measured using RT-PCR. Data are presented as the mean ± standard deviation. A student’s t-test was used for statistical analysis: *, *P* < 0.05. The assay was repeated three times

Compared with the non-PCOS group, the mRNA expression levels of *GLUT1*/*LDHA*/*PFKP* were significantly downregulated in the GCs obtained from patients with PCOS (*GLUT1*: 1.077 ± 0.10 vs. 0.734 ± 0.10; *LDHA*: 1.005 ± 0.04 vs. 0.641 ± 0.1; *PFKP*: 1.103 ± 0.08 vs. 0.723 ± 0.10) (Figs. 3A–C).

### Glucose metabolism in follicular fluid

**Fig. 4:**
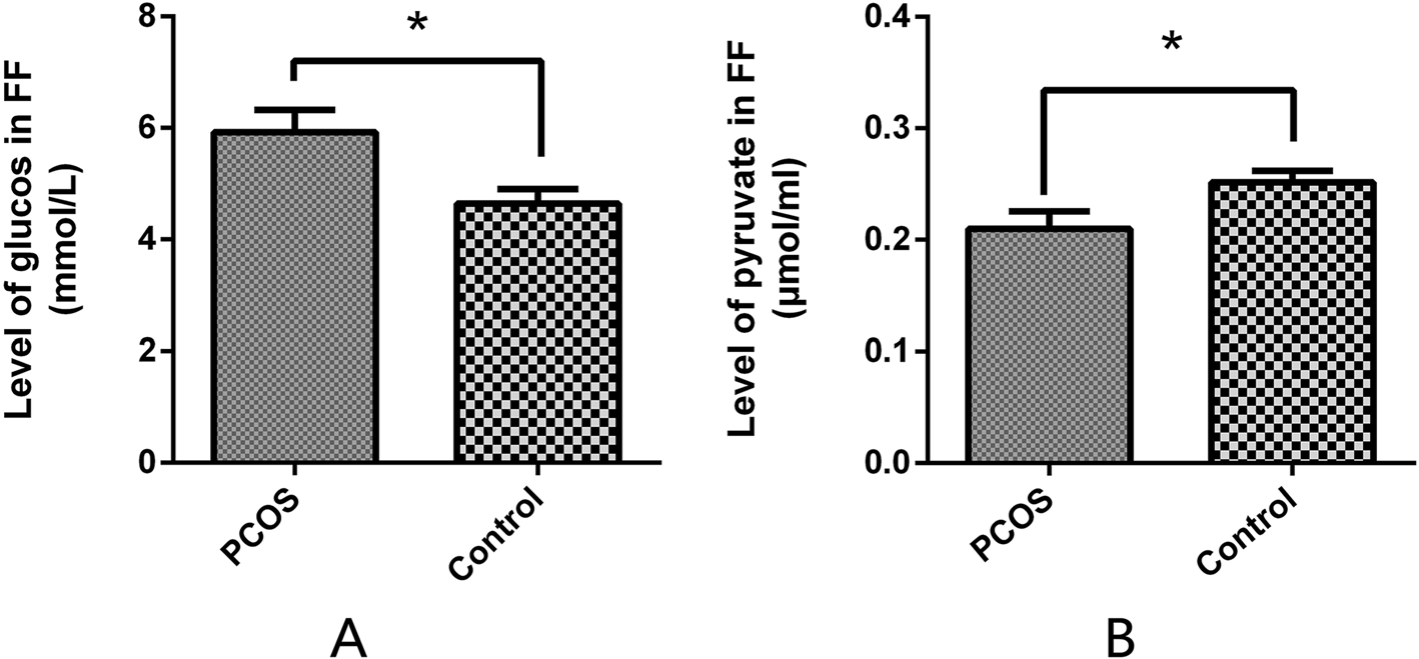
Abnormal glucose (**A**) and pyruvate (**B**) levels were measured in the follicular fluid of patients with and without polycystic ovary syndrome. Data are presented as the mean ± standard deviation. A student’s t-test was used for statistical analysis: *, *P* < 0.05. The assay was repeated three times

The glucose and pyruvate levels in the FF were tested and measured after GC isolation. Similar to the low expression pattern of the glycolysis-associated gene in the GCs from patients with PCOS, the levels of glucose were elevated (2.812 ± 0.33 vs. 5.505 ± 0.51 mmol/L) (Fig. 4A), accompanied by decreased levels of pyruvate (0.2347 ± 0.03 vs. 0.1673 ± 0.01 µmol/mL) (Fig. 4B).

## Discussion

A better understanding of molecular biology underlying human fertility plays a crucial role in enriching the knowledge in reproductive physiology and pathology. By unraveling the intricate mechanisms involved in fertility, researchers and healthcare professionals can develop more effective strategies for diagnosing, treating, and managing infertility [12–14]. Human GCs primarily rely on mitochondrial oxidative phosphorylation and glycolysis for energy production. In this study, we compared the characteristics of clinical IVF cycles between patients with and without PCOS. Consistent with the characteristics of PCO, the results showed that the body mass index, basal LH, T, and fasting blood glucose (FGB) levels in the PCOS group were higher than those in the non-PCOS group, whereas the FSH levels were lower. Furthermore, the mRNA levels of *GLUT1*/*LDHA*/*PFKP in GCs* were significantly downregulated, and the levels of MMPs and mtDNA were decreased, suggesting that mitochondria and glycolysis jointly participate in the energy metabolism of PCOS ovarian GCs. In addition, the GCs’ mitochondrial function in the PCOS group decreased compared with that in the non-PCOS group. In the PCOS group, the levels of MMP, ATP, and mtDNA decreased significantly, whereas the level of ROS increased significantly. These findings indicate that mitochondrial respiration and glycolysis both contribute to the energy metabolism of GCs. Mitochondrial dysfunction accompanied by abnormal glycolysis was observed in PCOS patients. The source of excessive ROS generation in PCOS might be impaired mitochondrial function. In addition, GCs derive their energy from mitochondrial respiration and glycolysis. Mitochondrial dysfunction accompanied by abnormal glycolysis in GCs during the development of follicles might be correlated with the low oocyte competence of PCOS.

PCOS is one of the most prevalent endocrine diseases in women of childbearing age and has a significant impact on reproductive function. Persistent follicular development and maturation disorders in the ovaries characterize this condition. Previous studies have shown that the oocytes collected from patients with PCOS undergoing IVF-ET are frequently of low quality, and the pregnancy outcomes are not optimal [9–11]. The cause of PCOS follicular maturation disorder and low oocyte development competence has not been fully understood. The potential responsible factors include nutrition, hormonal regulation, and environmental influence [15]. GC dysfunction and metabolic disorders have been shown to contribute to abnormal folliculogenesis in PCOS [16, 17]. In the present study, we showed that mitochondrial function significantly declined and was accompanied by increased ROS production in the GCs of patients with PCOS [18].

During the development, maturation, fertilization, and embryonic development of oocytes, a large amount of ATP is produced by the oxidative phosphorylation of mitochondria to provide energy. However, due to the limited efficiency of direct utilization of glucose by oocytes, pyruvate, a glycolysis pathway product of GCs, must be transported to the mitochondria of oocytes via the monocarboxylic acid transport system to serve as a substrate for the ATP energy production of oocytes. Our data showed that the MMP and ATP levels declined and ROS levels increased, indicating reduced mitochondrial function in the GCs of the PCOS group. Furthermore, excessive glucose in the FF and fasting blood glucose obtained from patients with PCOS was accompanied by the downregulated expression of glycolytic rate-limiting enzymes *PFKP*, *GLUT1*, and *LDHA*, indicating decreased glycolysis activity in the GCs. The overexpression of oxidative stress (OS) and the disorder of glucose metabolism in the ovarian microenvironment may influence the development potential of oocytes and early embryos.

Our study also demonstrated that the mtDNA genes of the GCs were downregulated in patients with PCOS. MMP is an important indicator of cell viability, whereas mtDNA is essential for maintaining mitochondrial function and cell growth. GCs respond excessively to OS during ovarian development, which can result in a decrease in the mtDNA copy number and MMP and even apoptosis. Compared with the non-PCOS group, the OS level significantly increased in patients with PCOS. In patients with PCOS, the increase in peroxidation injuries indicated by MDA and *8-OHDG* was accompanied by the compensatory elevated antioxidant damage index SOD. Abnormal mitochondrial function at the cellular level may affect the metabolic homeostasis of the whole body [9].

Previous evidence has suggested that GC dysfunction contributes to abnormal folliculogenesis in anovulation disease [16]. In PCOS, the mitochondrial dysfunction of GCs also affects oocyte maturation and development [18]. Accumulating evidence has indicated that insulin resistance is a significant mechanism underlying the development of PCOS. Consequently, there has been growing interest in the use of insulin-sensitizing agents, particularly inositol isoforms, owing to their favorable safety profile and demonstrated efficacy. The recognition of insulin resistance as a key factor in PCOS pathogenesis may facilitate the development of targeted therapeutic interventions aimed at improving insulin sensitivity [17, 19]. Furthermore, the development potential of oocytes with low mtDNA copy numbers is significantly reduced, thereby reducing blastocyst formation, which is consistent with the common symptoms of patients with PCOS, such as anovulation and infertility [20, 21]. GCs have two energy metabolism pathways—mitochondrial oxidative phosphorylation and glycolysis—involved in sustaining the stability of the internal environment of the follicle during the later stages of follicular development and maturation. The stable mitochondrial function promotes the transformation of the energy mode of the GCs to the glycolysis mode in the process of follicular development. The damage to the energy metabolism function of ovarian GCs may be one of the mechanisms responsible for the decline of oocyte development competence in patients with PCOS.

Our study revealed that the mitochondrial function and glycolytic activity of GCs are crucial for the production of high-quality oocytes and successful embryonic development. The presence of abnormal energy metabolism dysfunction in GCs may also contribute to the impaired folliculogenesis observed in ovulatory disorders in human reproduction. However, the mechanisms by which the HIF-1 activity affects energy metabolism switching in human primary granulosa cells, as well as the mitochondrial function underlying the decline in oocyte development competence in patients with polycystic ovary syndrome (PCOS), were not explored. Further investigations on this topic are needed.

## Conclusion

The current study has revealed that during folliculogenesis, patients with PCOS exhibit abnormal mitochondrial function and disordered glycolytic activity. In PCOS, an improvement in mitochondrial respiratory function and glucose metabolism might enhance embryonic competence. These results highlight the need for further research aimed at stabilizing mitochondrial function and promoting GC proliferation. Such interventions could potentially improve fertility outcomes in patients with PCOS and contribute to the development of more effective treatment strategies for these patients.

## Abbreviations

GCs: Granulosa cells
COH: Controlled ovarian hyperstimulation
ATP: Adenosine triphosphate
PCOS: Polycystic ovary syndrome
IVF: In vitro fertilization
IVF-ET: In vitro fertilization-embryo transfer
GnRH-a: Gonadotropin-releasing hormone agonist
FF: Follicular fluid
MMP: Mitochondrial membrane potential
ROS: Reactive oxygen species
MDA: Malondialdehyde
SOD: Superoxide dismutase
